# Concurrent Brucellosis and Q Fever Infection: a Case Control Study in Bamyan Province, Afghanistan

**DOI:** 10.5195/cajgh.2013.58

**Published:** 2014-01-03

**Authors:** Khwaja Mir Islam Saeed, Jamalludin Ahadi, Mohammad Nadir Sahak, Ahmad Farid Ghiasi, Rana Jawad Ashgar

**Affiliations:** 1 Afghanistan National Public Health Institute, Ministry of Public Health, Kabul, Afghanistan; 2 World Health Organization, Kabul, Afghanistan; 3 National Institute of Health, Islamabad, Pakistan

**Keywords:** Brucellosis, Q Fever, Afghanistan, Zoonoses

## Abstract

**Background:**

More than 500,000 people are affected by brucellosis each year while the incidence of Q fever is poorly recorded. Consistent outbreaks of brucellosis have been reported in Afghanistan, affecting social and economic life. This study aimed to determine the means of propagation of brucellosis and Q-fever and establish appropriate control measures for both.

**Methods and Materials:**

An outbreak of 1,317 cases of brucellosis and Q fever was investigated from May 2011 to the end of 2012 in Bamyan province of Afghanistan. A total of 100 cases were selected by random sampling with equal number of neighbor controls. Data were collected through structured questionnaire.

**Results:**

The average age was 30 years ±14 years. Of those sampled, 62% were female, 38% were male, and resided in three districts: Punjab, Yakawlang and Waras. Using multivariate analysis, being a housewife (OR=7.36), being within proximity of kitchens to barns (OR= 2.98), drinking un-boiled milk (OR= 5.26), butchering (OR= 3.53) and purchasing new animals in the last six months (OR= 3.53) were significantly associated with contraction of brucellosis and Q fever.

**Conclusion:**

Health educators should focus on families dealing with animals, especially on females. Pasturing, healthy milking, dunging, and slaughtering practices, along with use of safe dairy products should be the focus of preventive measures.

## Introduction

Brucellosis is caused by bacteria of the genus *Brucella*; species considered important agents for human disease are *B*. *Melitensis*, *B. Abortus* and *B.Suis.*^1^ It is an old disease with minimal mortality and remains the most common zoonotic disease worldwide.^2^ The incubation period for brucellosis is variable and difficult to ascertain; however, it is usually 5–60 days and occasionally several months after exposure.^3^

The World Health Organization (WHO) estimates that 500,000 brucellosis cases occur each year worldwide, 45,000 of which occur in the Eastern Mediterranean Region. For every case diagnosed, there are four cases that go undetected.^4^ Brucellosis is a major public and animal health problem in many regions of the world, particularly where livestock are a major source of food and income. Despite control programs, it remains endemic in most developing countries.^5^ The WHO considers brucellosis to be a neglected zoonosis because, despite its widespread distribution and effects on multiple species, it is not prioritized by national and international health systems.^6^ Human cases continue to occur following traditional use of raw milk products and close contact with infected animals.

According to a case-control study in Saudi Arabia, the consumption of unpasteurized dairy products derived from sheep and goats as well as assistance in animal parturition were greatly associated with brucellosis.^8^ In a similar study in Yemen, being a farmer, shepherd, or microbiologist, and drinking fresh milk and buttermilk were significantly associated with brucellosis. Socio-economic and educational factors were also independent risk factors.^9^ In Iran, the same type of study revealed that significant risk factors for infection were related to the existence of another case of brucellosis in the home and consumption of unpasteurized dairy products.^10^

Investigations carried out in Africa support these findings. Risk factors, such as assisting parturition during abortion and living in close proximity to other households affected with brucellosis, were identified.^11^ Another study, which was conducted to identify the potential risk factors for human brucellosis infection in Samarqand, Uzbekistan showed that brucellosis was highly associated with contact with aborted animals, slaughtering/butchering animals in the household, consumption of raw milk, and being in a family that had brucellosis sharing the same exposure.^13^ In a study from Kyrgyzstan, results of multivariate analysis indicated that brucellosis was associated with exposure to aborted farm animals in the household and consumption of home-made milk products obtained from bazaars or neighbors. Knowledge of the mode of brucellosis transmission appeared to be protective against disease transmission.^14^

Alongside other endemic communicable diseases, Query Fever (Q-Fever) is also common in developing countries. Q fever is caused by the bacteria *Coxiellaburnetii* and can affect the lungs, liver, heart, and other parts of the body. It is found around the world and affects sheep, goats, cattle, dogs, cats, birds, rodents, and several other types of animals. Infected animals shed these bacteria in birth products, feces, milk, and urine. Humans usually contract Q fever by breathing in contaminated droplets released by infected animals. Drinking raw milk has also caused infection in rare cases. People at highest risk for this infection are farmers, laboratory workers, sheep and dairy workers, and veterinarians.^15^ Initially described in Australia in 1937, it is prevalent in southern France and Spain and is the second most common cause of community-acquired pneumonia, causing 5–8% of the endocarditis cases. Infected dairy goat farms are believed to be the source of the outbreak among humans.^16^

Few studies have been conducted in Afghanistan regarding the prevalence and risk factors of communicable diseases. Brucellosis has been counted among the endemic diseases in Afghanistan.^17^ In a global incidence report, Afghanistan is categorized in the group of 2–8/1,000,000;^18^ whereas according to a OIE (World Organization for Animal Health) report in 2005, the incidence of brucellosis was 3.8 per millionin country.^19^ Moreover, outbreaks of brucellosis have consistently been detected and investigated in Bamyan province, especially Punjab district. In August 2007 in Punjab district of Bamyan province, 35 cases were detected. Five samples were collected, and 3 were positive for Brucellosis. In September 2007 in Punjab district of Bamyan province, 43 cases were detected, 10 samples were collected, and 8 samples were positive. In June 2008 in Punjab district of Bamyan province, 10 cases were detected, 4 samples were collected, and all were positive for brucellosis.^20^ However, data regarding the burden of Q-Fever are not published in Afghanistan, but extrapolation of the incidence rate for Q fever is estimated at 38 per 1,000,000 nationally.^21^ A report from a hospital in the UK shows that some members of the army who came back from Afghanistan were diagnosed with Q-Fever.^22^ It is confirmed by another report from the US army, which found that after the deployment of thousands of American troops in Afghanistan since 2001, some common and chronic infections have persisted, including Q fever, brucellosis, and parasitic infections.^23^

The co-infection of both brucellosis and Q-Fever is poorly recorded in the literature. The aim of this study is to investigate the outbreak and determine the risk factors associated with brucellosis and Q-Fever acquisition in the central province of Bamyan and to provide recommendations for control of brucellosis in Bamyan Province.

## Methods and Materials

### Study area

On May 29^th^, 2011, the mobile health unit and district health officer at Yakawlang District reported the suspected outbreak of brucellosis to the provincial public health directorate in Bamyan province. The outbreak was in the border area between two districts of Yakawlang and Punjab, separated by only a hilly mountain. The residents of these districts are primarily livestock keepers who practice traditional pastoralism and follow a semi-nomadic lifestyle. A team consisting of an epidemiologist, a medical doctor, a veterinarian, and a laboratory technician was dispatched to the area.

### Cases definitions and blood sampling

Operational case definition was developed prior to data collection. Those who had recurrent or continuous fever, joint pain/swelling, and general body malaise or backache in the area were considered to be suspected cases while those who exhibited the above symptoms and had contact with animals were thought as probable cases. Finally, those suspected and/or probable cases that were verified by laboratory testing (Rose Bengal Test) were considered to be confirmed cases. Initially in June 2011, a total of 147 subjects were line listed; blood was sampled and tested for brucellosis from 39 suspected cases at the district level. Twenty-eight samples were shared with the Food and Agriculture Organization supported laboratory at the Ministry of Agriculture, Irrigation and Livestock (MAIL) to test for brucellosis and Q-fever.

### Study design

As part of the outbreak investigation, a case control study was conducted to identify the associated factors for brucellosis and Q-Fever in order to formulate health education and preventive measures. Cases were defined as those showing at least two of the following clinical features: recurrent or continuous fever, sweating, joint pain, joint swelling, general body malaise, or backache. A total of 100 cases were identified. For every case, a control was selected randomly, irrespective of exposure from neighbors. Cases were given appropriate treatment and referred to hospital if necessary. Health education sessions were conducted during investigation and cases were managed clinically at the district hospital.

### Data collection and analysis

A questionnaire asking for demographics and risk factors was developed by research team and used to collect data from both cases and controls. Data collection was completed in July 2011 and was entered in Epi Info 3.5.3. After cleaning, the data was analyzed using IBM Statistical Package for the Social Sciences (SPSS) statistical software version 20.^24^ Data were analyzed by time, person and place along with clinical information. Logistic regression analysis was used to analyze the data at univariate and multivariable levels.

## Results

### Descriptive Analysis

All age groups within the population were affected by the infection; however, those who were 15 to 30 years of age were most commonly affected compared to the other age groups. Of 39 blood samples collected initially and tested using the Rose Bengal test at the local level, 59% (n=23) of the samples from Punjab District Hospital and 16 samples from Yakawlang district hospital tested positive for brucellosis.

**Figure 1:**
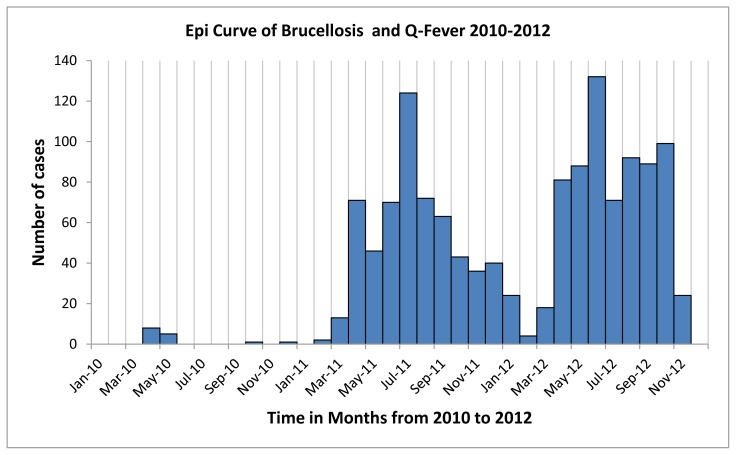
Epidemic curve of brucellosis and Q fever outbreak in Bamyan province

Of the 28 samples, which were tested in the Ministry of Agriculture laboratory using the Rose Bengal Test, all were positive for brucellosis. After conduction of a laboratory tests for Q-Fever in MAIL, it was found that 96.4% (n=27) samples were also positive for Q-Fever on PCR. A wide range of clinical signs and symptoms including fever (100%), general body pain (96%), back pain (93%), joint pain (92%), chills (78%), sweating (86%), anorexia (95%), and tremor (66%) were common among cases. The majority had contact with animals and/or their products. The team was of the opinion that this was potentially the beginning of a more extensive outbreak. This was confirmed after the first round of investigation by a case-control study conducted from July to September 2011. Line listing continued until the December 31, 2012. 1,317 total cases were line listed until November 31, 2012. The updated epidemic curve, which includes the cases for 2010 to 2012, is reflected in Figure 1. The first peak is due to the outbreak while the second peak might be due to delivery of medical supplies for treatment of disease in the province.

The ages of cases during the outbreak varied from one year to 75 years, with an average age of 30 years. 62% were female and 38% (498) were male. The mean age of the cases was 29.5 years ± 14 SD and the mean age of the controls was 25.7 ± 16.6 years. However, this difference in age was not statistically significant. Education status of both cases and controls was low, with 69% being illiterate and 49% housewives. Out of the study participants, 83% did not have knowledge about brucellosis and Q fever, while 96% owned animals at their home. 57% had sick animals within a one-year period. Almost 80% had less than 5 cows. 82% had less than 30 sheep and less than 8 goats at their stables. More than 90% of them had contact with animals, including feeding, watering, pasturing, milking, dunging, slaughtering, and butchering. More than 90% used at least one of the following animal products: butter, cream, milk, yogurt, or other local products. Approximately 93% did not wash their hands with soap after handling animals. Table 1 describes the distribution of demographic and socio-economic variables as a whole among study participants.

### Univariate Analysis

There was a significant difference among age groups. Those aged less than 15 years were used as a reference group. It seems that the higher age groups were at higher risk than the lowest age group. Similarly, there was a significant difference between cases and controls by sex, with an odds ratio of 2.40 and 95 % confidence interval of 1.37 to 4.44. Level of education (as a proxy for socio economic status) showed significant differences between illiteracy and secondary education (OR=2.27 and 95% CI=1.13 – 4.58). This difference was not significant between primary and secondary education. There was no significant difference between employees of the government and students or between those owning a small business and farmers. However, those working as housewives were significantly different from others with an odds ratio of 8.32 and 95% CI of 3.40 to 20.43. There was no association with having or not having knowledge of diseases among cases and controls. Table 2 shows the association of socio-demographic factors with brucellosis and Q fever.

We found a significant difference among cases and controls with respect to having sick animals at home in the last six months with an odds ratio of 3.89 and 95 % CI of 2.14 to 7.05. Likewise, there was a significant association between those who had more than 5 cows and more than 30 sheep with an odd ratios of 3.03 (95% CI = 1.55 – 7.07) and 2.86 (95% CI = 1.40 – 5.90) respectively. There was no association between the number of goats between cases and controls. There were significant differences among cases and controls with respect to pasturing (OR= 2.63 and 95% CI = 1.35 – 5.10), milking (OR= 3.04 and 95% CI = 1.71 – 5.42), dunging (OR= 3.81 and 95% CI = 2.10 – 6.96), butchering (OR= 3.03 and 95% CI = 1.70 – 5.40), and assisting deliveries in animals (OR= 3.80 and 95% CI = 2.11 – 6.85). Approximately all cases and control were using different dairy products and it was not possible to test the difference among groups. Cases were 3.82 (95% CI = 2.12 – 6.90) times more likely to be exposed to aborted materials as compared to controls. There was a significant difference in the frequency of boiling milk between cases and controls. The odds ratio of drinking milk without boiling was 7.04 (95% CI = 3.43 – 14.46) times higher among cases as compared to controls. Cases had 2.12 (95% CI = 1.19 – 3.77) and 2.27 (95% CI = 1.10 – 4.65) times more odds of exposure in slaughtering animals or living in close proximity with animals at home as compared to control groups. The cases had 4.55 (95% CI = 2.15 – 9.61) times more odds of living within 10 meters of their animals as compared to controls. Table 3 shows detailed information regarding the association of these risk factors to contraction of Brucellosis and Q fever.

### Multivariate Analysis

Multiple logistic regression analyses method was used to adjust for confounding. We used the biological as well as statistical significance (p ≤ 0.25) as criteria for inclusion in our model. Table 4 shows multivariate analysis results with adjusted OR and 95 % Confidence Intervals (CI).

According to multivariate analysis, housewives are 7.36 (95% CI = 3.05 – 17.78) times as likely to be infected compared to students and government employees. Infected individualswere 3 times (95% CI = 1.21 – 7.35) as likelyof living in close proximity to animals (>10 meter) as compared to those controls. In addition, we found significant association with drinking un-boiled milk (OR= 5.26, 95% CI = 2.30 – 12.02), being a butcher (OR = 3.53, 95% CI = 1.56 – 8.10), and purchasing new animals in the last six months (OR= 3.53, 95% CI = 1.42 – 8.53) with infection of brucellosis and Q fever.

## Discussion

Concurrent infection of Brucellosis-Q fever has been detected in an outbreak in which all cases have been in contact with animals and their products. According to the findings of our study using univariate analysis, gender and age group is significantly associated with brucellosis and Q fever infections. We expected that males, due to their professions, should have been affected more often by brucellosis than females.^25^ This has been supported by a sero-prevalence study of human brucellosis in Turkey in which a statistically significant correlation found between seropositivity and age, sex, and consuming fresh cheese and cream made from un-boiled milk.^12^ In addition, illiteracy was another significant risk factor for infection at this level. This may be due to less concern with being healthy or lower awareness of preventive and control measures. Housewives in Afghanistan are busy with the management of animal products, and this maybe the reason for the significance of the relationship between this occupation and infection.

More important is contact with aborted materials, which is thought to be the source of infection. The study showed significant association with assisting deliveries and brucellosis infection. The finding that contact with livestock during parturition is a strong risk factor for brucellosis is consistent with results from other studies, which demonstrate an increased risk in association with assisted parturition.^26^ Protective measures such as using gloves, gowns and masks should be used while touching these materials, and the product should be discharged safely. Hands should be washed with soap after handling animals. Before purchasing animals, buyers should ensure that the animals are healthy. The stables of animals should be away from residential areas or at least substantial distance from rooms and kitchens.

However, using multivariate analysis, being a housewife, having a kitchen in close proximity to animals, drinking and using un-boiled milk, involvement in butchering activities, and purchasing new animals in last six months were significantly related to infection. It seems that housewives are in more contact with animals and animal products compared to other groups. This should be a focus for health education activities. In addition, the boiling of milk before using or drinking should be promoted, if not enforced. The butchers and slaughterers should have more information on disease to avoid unprotected contacts with animals. The new animals’ movement and marketing should be managed by veterinary services in the province as well as the country in general.

The approach to control, prevention, or eradication of brucellosis in a country or region will depend on many factors, such as the level of infection in the herds or flocks, type of husbandry, economic resources, public health impacts, and potential international trade implication.^7^ The implementation of control measures, including sustained enhanced surveillance and required case management activities, is expected to pose a challenge to the national public health system. There is a need to expand and enhance the surveillance system and strengthen collaborations and coordination with veterinary services. Effective control of brucellosis requires a long-term commitment from many governmental agencies and assistance from international animal and human health organizations. An educational program is needed for high risk groups for zoonosis about the prevention of infection.

## Figures and Tables

**Table 1:** Frequency distribution of the socio-demographic characteristics of study participants in Bamyan Province- Afghanistan in July 2011 (N=200)

SociodemographicVariables	N (%)
**Age (years)**	
<15	53 (26.5)
15–30	72 (36.0)
30–45	46 (23.0)
>45	29 (14.5)
**Sex**	
Male	77 (38.5)
Female	123 (61.5)
**Education Status**	
Illiterate	138 (69.0)
Primary Education	18 (09.0)
Secondary Education	44 (22.0)
**Occupation**	
Government employee & student	36 (28.0)
Small Business holder	25 (12.5)
Farmers	41 (20.5)
Housewife	98 (49.0)
**Knowledge of Brucellosis and Q fever**	
Yes	34 (17.0)
No	166 (83.0)
**Residence (Districts)**	
Panjab	150 (75.0)
Waras	50 (25.0)

**Table 2:** Statistical analysis of the risk factors (Socio-economic) associated with Brucellosis and Q fever in Bamyan Province in July 2011

Variable	Cases	Controls	OR	95 % CI
**Age**				
<15	16	37	1	Reference
15 – <30	42	30	3.23	1.52 – 6.85
30 – <45	27	19	3.28	1.43 – 7.53
≥45	15	14	2.47	0.97 – 6.31
**Sex**				
Male	51	49	1	Reference
Female	49	51	2.4	1.37 – 4.44
**Education Status**				
Illiterate	78	60	2.27	1.13 – 4.58
Primary Education	12	12	0.87	0.27 –2.78
Secondary Education	10	28	1	Reference
**Occupation**				
Housewife	69	29	8.32	3.40 – 20.43
Farmer	17	24	2.48	0.91 – 6.75
Small Business	6	19	1.1	0.33 – 3.69
Student	8	28	1	Reference
**Knowledge of Diseases**				
Yes	16	18	1	Reference
No	84	82	0.86	0.41 – 1.81

**Table 3:** Statistical analysis of behavioral risk factors associated with Brucellosis and Q fever in Bamyan Province in July 2011

Variables	Cases	Controls	OR	95 % CI
Having a sick animal in the last 6 months				
No	27	59	1.00	Reference
Yes	73	41	3.89	2.14–7.05
Types and number of animals				
Cattle Group				
≤ 4	71	89	1.00	Reference
> 4	29	11	3.03	1.55–7.07
Sheep Group				
≤ 30	70	87	1.00	Reference
> 30	30	13	2.86	1.40–5.90
Goat Group				
≤ 8	79	58	1.00	Reference
> 8	21	14	1.63	0.77–3.40
Type of activities in animal husbandry				
Pasturing				
No	23	65	1.00	Reference
Yes	17	35	2.63	1.35–5.10
Milking				
No	67	40	1.00	Reference
Yes	33	60	3.04	1.71–5342
Dunging				
No	75	44	1.00	Reference
Yes	25	56	3.81	2.10–6.96
Butchering				
No	61	34	1.00	Reference
Yes	39	66	3.03	1.70–5.40
Assisting Delivery				
No	70	38	1.00	Reference
Yes	30	62	3.63	2.02–6.52
Contact with Aborted Products				
No	29	61	1.00	Reference
Yes	71	39	3.82	2.12–6.90
Boiling of milk before drinking				
Yes	51	88	1.00	Reference
No	49	12	7.04	3.43–14.46
Slaughtering Animals				
No	32	50	1.00	Reference
Yes	68	50	2.12	1.19–3.77
Living in close proximity to animals				
No	9	32	1.00	Reference
Yes	91	68	2.27	1.10–4.65
Distance from kitchen >10 meters				
Yes	11	36	1.00	Reference
No	89	64	4.55	2.15–9.61
Purchasing new animals in last 6 months				
No	70	88	1.00	Reference
Yes	30	12	3.14	1.50–6.58

**Table 4:** Multivariate analysis of risk factors associated with Brucellosis and Q fever in Bamyan Province

Variables	Adjusted OR	95% CI	P-value
Working status/Job category			
Housewives	7.36	3.05–71.78	>0.001
Farmers	1.19	0.42–3.34	0.738
Students/govt. employees	1.00	Reference	-
Distance from kitchen			
<10 meters	2.95	1.21–7.35	>0.05
>10 meters	1.00	Reference	-
Drinking milk after boiling			
No	5.26	2.30–12.02	>0.001
Yes	1.00	Reference	-
Involvement in butchering activities			
Yes	3.53	1.56–8.10	>0.05
No	1.00	Reference	-
Purchasing new animals in the last 6 months			
Yes	3.53	1.42–8.53	>0.05
No	1.00	Reference	-
